# Spatial-temporal drivers and incidence heterogeneity of hemorrhagic fever with renal syndrome transmission in Shandong Province, China, 2016–2022

**DOI:** 10.1186/s12889-024-18440-x

**Published:** 2024-04-13

**Authors:** Qing Duan, Yao Wang, Xiaolin Jiang, Shujun Ding, Yuwei Zhang, Mingxiao Yao, Bo Pang, Xueying Tian, Wei Ma, Zengqiang Kou, Hongling Wen

**Affiliations:** 1 Infectious Disease Prevention and Control Section, Shandong Center for Disease Control and Prevention, Jinan, 250014 China; 2 Department of Epidemiology, School of Public Health, Cheeloo College of Medicine, Shandong University, Jinan, 250012 China; 3 Department of Microbiological Laboratory Technology, School of Public Health, Cheeloo College of Medicine, Shandong University, Jinan, 250012 China; 4 Ministry of Research and Education, Shandong Center for Disease Control and Prevention, Jinan, 250014 China; 5 Infection Disease Control of Institute, Shandong Center for Disease Control and Prevention, Shandong Provincial Key Laboratory of Infectious Disease Prevention and Control, Jinan, 250014 China

**Keywords:** Hemorrhagic fever with renal syndrome, Spatial-temporal multicomponent model, Transmission driver, Influencing factor

## Abstract

**Background:**

Hemorrhagic fever with renal syndrome (HFRS) signals a recurring risk in Eurasia in recent years owing to its continued rise in case notifications and the extension of geographical distribution. This study was undertaken to investigate the spatiotemporal drivers and incidence heterogeneity of HFRS transmission in Shandong Province.

**Methods:**

The epidemiological data for HFRS, meteorological data and socioeconomic data were obtained from China Information System for Disease Control and Prevention, China Meteorological Data Sharing Service System, and Shandong Statistical Yearbook, respectively. The spatial-temporal multicomponent model was employed to analyze the values of spatial-temporal components and the heterogeneity of HFRS transmission across distinct regions.

**Results:**

The total effect values of the autoregressive, epidemic, and endemic components were 0.451, 0.187, and 0.033, respectively, exhibiting significant heterogeneity across various cities. This suggested a pivotal role of the autoregressive component in propelling HFRS transmission in Shandong Province. The epidemic component of Qingdao, Weifang, Yantai, Weihai, and Jining declined sharply at the onset of 2020. The random effect identified distinct incidence levels associated with Qingdao and Weifang, signifying regional variations in HFRS occurrence.

**Conclusions:**

The autoregressive component emerged as a significant driver in the transmission of HFRS in Shandong Province. Targeted preventive measures should be strategically implemented across various regions, taking into account the predominant component influencing the epidemic.

**Supplementary Information:**

The online version contains supplementary material available at 10.1186/s12889-024-18440-x.

## Introduction

Hemorrhagic Fever with Renal Syndrome (HFRS) is a contagious disease transmitted by various families of Hantaviruses (HTNV), primarily carried and spread by multiple rodent species. Transmission to humans occurs through contact with rodent droppings, urine, and saliva, or inhalation of aerosols containing these substances [1, 2]. HTNV, with a globally distribution, pose a significant public health threat, leading to over 150,000 reported cases of HFRS worldwide annually, with the majority concentrated in Eurasia [3]. China has been disproportionately affected by HFRS, consistently representing over 90% of the global cases [4]. Despite a substantial reduction in HFRS incidence since the 1990s, attributed to comprehensive interventions like effective rodent control, environmental management, and vaccination, recent years have witnessed a resurgence of HFRS risk in China [4]. This resurgence is marked by an expanded geographical distribution and a sustained increase in reported cases [5, 6]. Given these developments, it is imperative to investigate the spatiotemporal transmission drivers of HFRS in different regions. Such an analysis would enable the implementation of targeted preventive measures and facilitate adjustments in resource allocation for the effective control of HFRS.

Prior investigations have delved into the spatial patterns and spatiotemporal clusters of HFRS cases, employing autocorrelation analysis and space scan analysis [7, 8, 9, 10]. These studies have successfully identified clustering areas at various scales [7, 8]. Moreover, research efforts have been dedicated to probing into the delayed and interaction effects of meteorological factors on HFRS incidence [9, 10]. Nonlinear techniques have also been applied to assess the temporal patterns of infectious diseases incidence, providing insights for predicting future occurrences [11, 12, 13]. Despite the valuable contributions of these studies in establishing a fundamental understanding of environmental variability for disease prevention, they often aggregated multiple cities or provinces in their studies. This oversight has obscured the inherent heterogeneity among cities, limiting the information available for the formulation of effective prevention and control measures for HFRS, as well as for the allocation of healthcare resources. Additionally, while previous research considered the seasonal pattern of HFRS incidence, it has not thoroughly explored the influence of neighboring regional epidemics on the ongoing local outbreak.

The spatial-temporal multicomponent model proposed by Held et al. is based on a branching process with immigration and Poisson offspring, which has been extended to allow for seasonality and overdispersion [14]. This model has been proved that is well suited to capture space-time dependence caused by the spatial spread of a disease over time [15]. The statistical model is constructed through an additive decomposition of disease incidence into three distinct components. Specifically, it comprises an endemic component and two epidemic components, each incorporating explicit autoregressions on the number of cases from the preceding time-point. Notably, one of the epidemic components is region-specific, considering the number of cases within the same geographical area. Meanwhile, the other epidemic component encompasses disease counts from different regions, thereby capturing the spatiotemporal spread of the disease.

In this study, we shifted our focus from the conventional exploration of associations between meteorological, socioeconomic factors, and HFRS incidence. Instead, we treated these external factors as covariates for model optimization. Subsequently, we conducted an analysis of spatiotemporal transmission dynamics within each specific region to identify key drivers. Our study contributed to the development of effective prevention and control measures and the optimization of healthcare resource allocation for HFRS.

## Materials and methods

### Study area

**Fig. 1:**
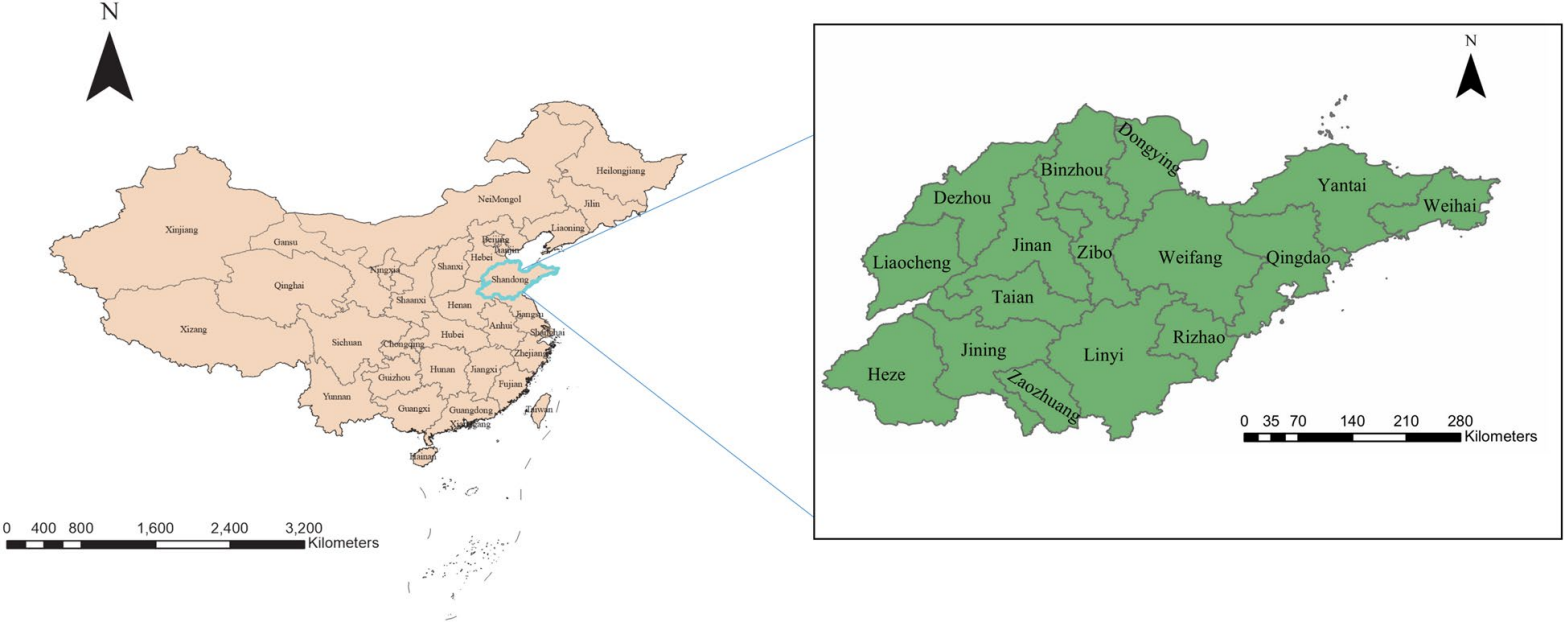
The geographical location of Shandong Province in China

This study area encompasses the entirety of Shandong Province, the northernmost coastal province in Eastern China, situated between latitudes 34°22.9′ N-38°24.01′ N and longitudes 114°47.5′ E-122°42.3′ E (Fig. 1). Shandong Province comprises two sub-provincial cities, namely Jinan and Qingdao, along with fourteen prefecture-level cities, including Binzhou, Dezhou, Dongying, Heze, Jining, Liaocheng, Linyi, Rizhao, Taian, Weifang, Weihai, Yantai, Zaozhuang, and Zibo. As of 2022, Shandong Province spans an area of approximately 0.1558 million square kilometers, boasting a population exceeding 100 million.

### Data collection

The weekly reported cases of HFRS spanning from January 1, 2016 to December 31, 2022, were obtained from the China Information System for Disease Control and Prevention (CISDCP). The diagnostic criteria for HFRS remained consistent throughout the entire study period.

The daily meteorological data for cities throughout the same period, encompassing mean temperature (°F), maximum temperature (°F), minimum temperature (°F), mean speed of gustiness (meters/second), maximum speed of gustiness (meters/second), relative humidity (%), hours of sunshine (hour) and wind speed (meters/second), were sourced from the China Meteorological Data Sharing Service System (http://data.cma.cn/). The city-level socioeconomic data, including population density, proportion of primary industry and Gross Domestic Product (GDP) per capita, were collected from the Shandong Statistical Yearbook (http://tjj.shandong.gov.cn/col/col6279/index.html).

### Spatial-temporal multicomponent model

This model additively decomposes disease risk into three components: the epidemic component, which captures transmission from other regions and reflects the influence exerted by neighboring units on the target unit; the endemic component, which captures exogenous factors such as seasonality, long-term trends, sociodemographic factors, and climatic factors, reflecting the local risk of the epidemic; and the autoregressive component, which reflects the impact of the past epidemics on the outcome of the present. The formula for this model is as follows:

$${\mu }_{i,t}={e}_{i,t}{v}_{i,t}+{\lambda }_{i,t}{Y}_{i,t-1}+{\theta }_{i,t}\sum _{i\ne j}({W}_{i,j}{Y}_{i-1,j})$$

$$\text{log}\left({v}_{i,t}\right)={\alpha }_{0}+{\alpha }_{i}+{\beta }_{t}+{z}_{i,t}^{T}\alpha +{S}_{eff}$$

$$S_{eff}=\left\{\sum_{S=1}^S\left[K_s\text{sin}\left(\varphi_st\right)+\delta_s\text{cos}\varphi_st\right]\right\}$$

$$\text{log}\left({\theta }_{i,t}\right)={\beta }_{0}+{\beta }_{i}+{k}_{i,t}^{T}\beta$$

$$\text{log}\left({\lambda }_{i,t}\right)={\gamma }_{0}+{\gamma }_{i}+{\mu }_{i,t}^{T}\gamma$$

Where ${Y}_{i,t}$ represents the disease count in the geographical region *i* = 1, …, *I* at time t = 1, …, *T*. ${W}_{i,j}$ is the neighborhood weights. ${Y}_{i,t}$ is a negative binomial distributed with mean E $({Y}_{i,t})$ = ${\mu }_{i,t}$. ${e}_{i,t}$ is the offset of region *i* over time *t*. In this study, we set the population proportion of different regions as the offset [16, 17]. ${v}_{i,t}$, ${\lambda }_{i,t}$and ${\theta }_{i,t}$ are the endemic component, autoregressive component, and epidemic component, respectively. ${\gamma }_{0}$, ${\alpha }_{0}$, and ${\beta }_{0}$ are component-specific intercepts. The random effects ${\alpha }_{i}$, ${\gamma }_{i}$, and ${\beta }_{i}$ are assumed to come from a normal distribution with a mean of zero. *S*_*eff*_ and *β* are the seasonal effect and long-term trend, respectively. ${z}_{i,t}^{T}$, ${\mu }_{i,t}^{T}$, and ${k}_{i,t}^{T}$ are the component-specific covariable matrix. Akaike information criterion (AIC) were adopted to choose the models and covariates.

### Statistical analysis

To mitigate multicollinearity, Spearman correlation analysis was performed to evaluate the correlation among covariables. Variables demonstrating a strong correlation (*r* > 0.5) were excluded from the final model simultaneously. The geographical location of the study area and the distribution of HFRS incidence were visualized using ArcGIS version 10.5 software (ESRI Inc., Redlands, CA, USA). Spatial-temporal multicomponent models were constructed at the city level with a weekly scale using R version 4.0.2 software and R package “surveillance. A significance level of *P* < 0.05 was employed for all tests.

## Results

### Descriptive results

**Fig. 2:**
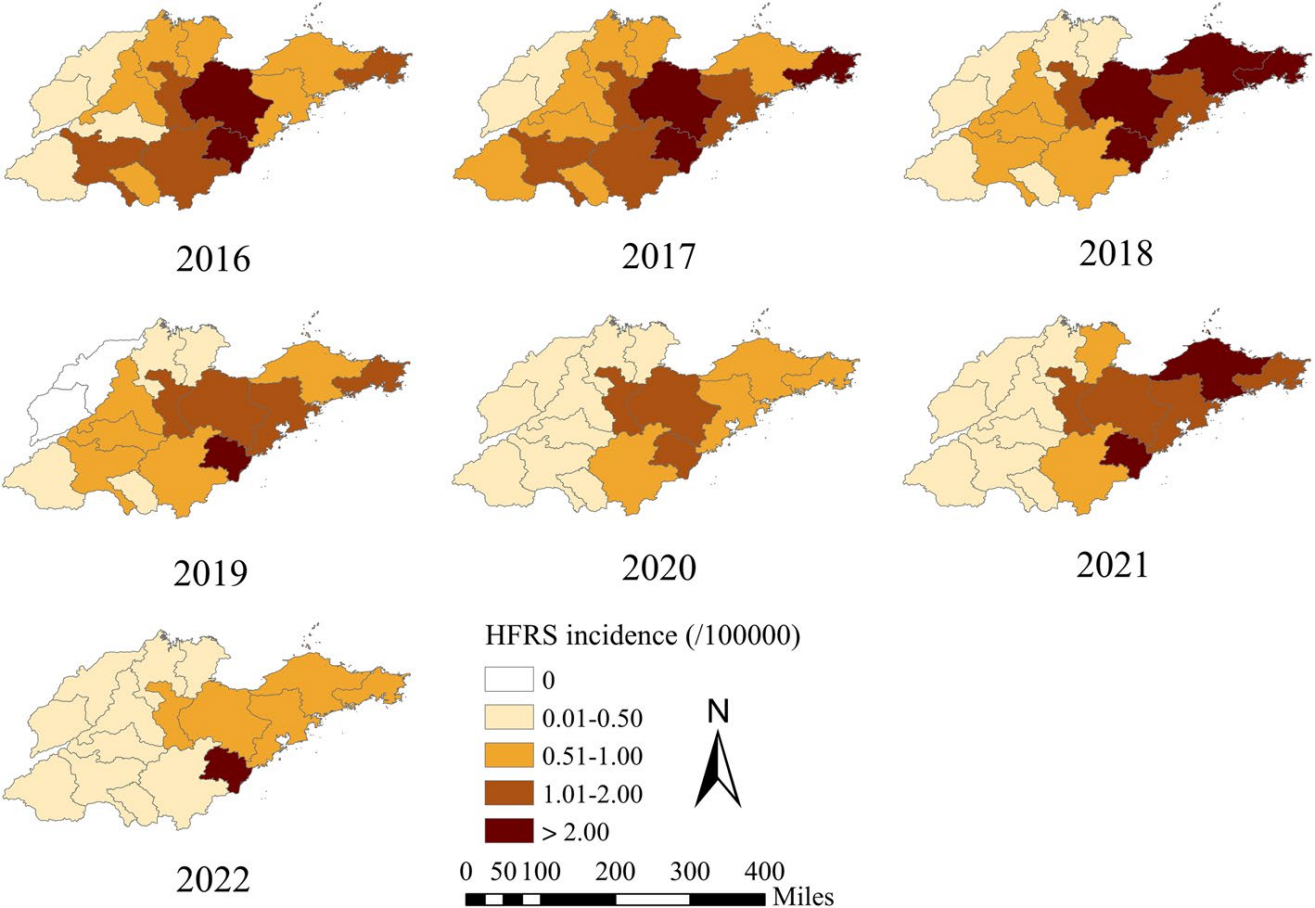
The incidence rate of HFRS in sixteen cities of Shandong Province, China, 2016–2022

**Fig. 3:**
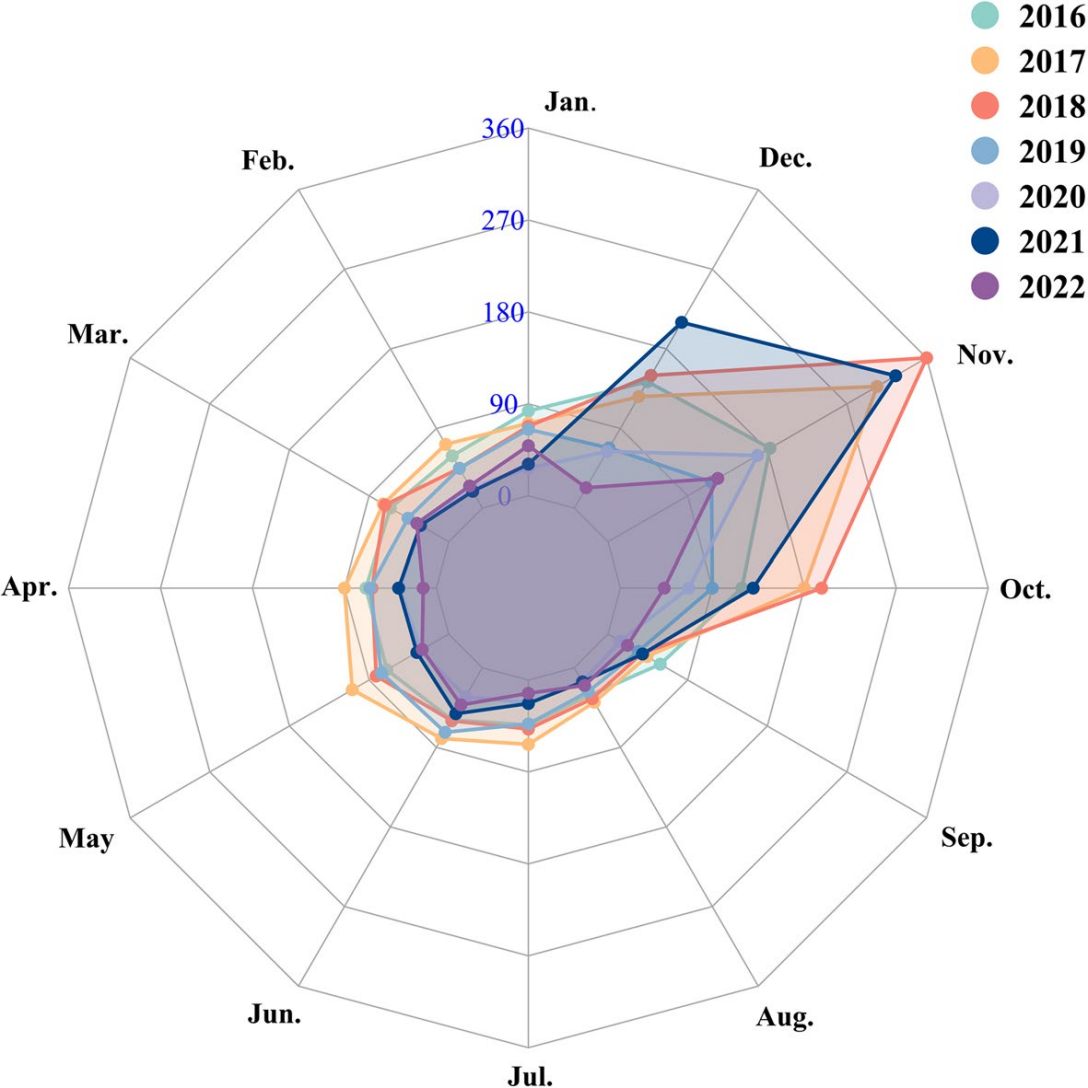
Monthly distribution of HFRS in Shandong Province, China, 2016–2022

Over the period of 2016–2022, Shandong Province reported 6,121 cases of HFRS, with incidence rates ranging from 1.259 per 100,000 to 0.433 per 100,000. The peak of the HFRS epidemic occurred in 2017, followed by a gradual decrease from 2019 (Fig. 2). Regions with a high incidence rate of HFRS were predominantly situated in central and eastern Shandong Province (Fig. 2). Notably, there was evident seasonality in the occurrence of HFRS over the seven-year period in Shandong Province (Fig. 3). Relatively fewer cases were reported in July, August and September compared to other months (Fig. 3).

### The spatial-temporal multicomponent model

**Fig. 4:**
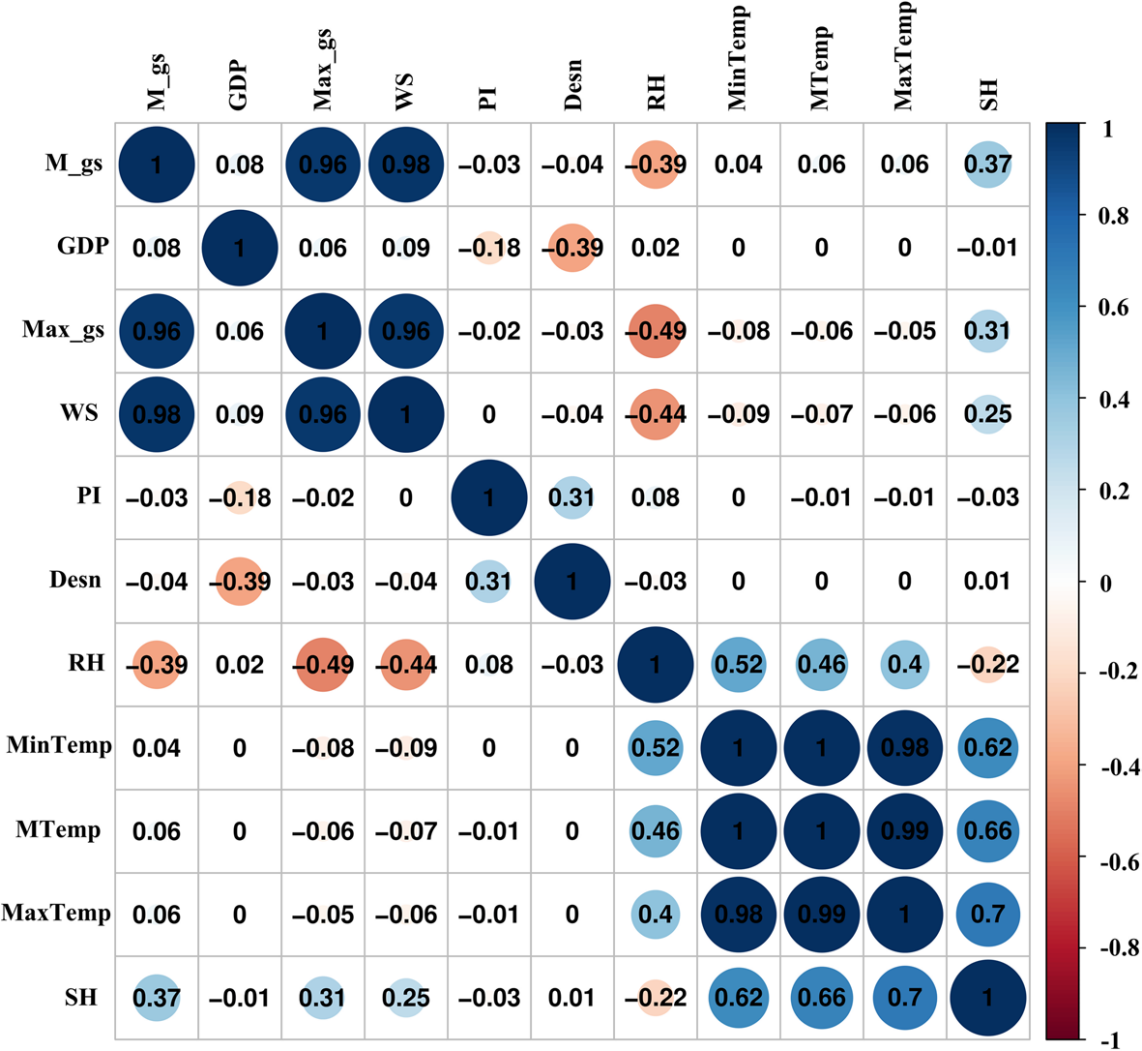
Correlation of meteorological and socioeconomic variables. PI: proportion of primary industry; MeanTemp: weekly mean temperature; MaxTemp: weekly maximum temperature; MinTemp: the weekly minimum temperature; M_gs: mean weekly speed of gustiness; Max_gs: weekly maximum speed of gustiness; Desn: population density; GDP: Real Gross Domestic Product per capita; RH: weekly mean relative humidity; SH: mean weekly hours of sunshine; WS: mean weekly wind speed

The model, which combined negative binomial distribution and power-law weights, was selected as the basic model based on AIC values (Table 1). Subsequently, covariables were independently integrated into the determined model to identify which factors were retained in the final model. The results showed that weekly mean temperature (AIC = 113291.63), mean speed of gustiness (AIC = 13263.96), maximum speed of gustiness (AIC = 13266.44) and GDP per capita (AIC = 13185.85) contributed to optimizing the previously selected model (Table 1). Considering AIC and collinearity (Table 1; Fig. 4), the final model included only GDP per capita, weekly mean temperature and maximum speed of gustiness (Table 1).

**Table 1:** Spatial-temporal multicomponent model of HFRS epidemic in Shandong Province from 2016 to 2022

Model	Autoregressive component (95%CI)	Epidemic component (95%CI)	Endemic component (95%CI)	γ_cov_ (95%CI)	AIC
Poisson distribution + first-order	0.461 (0.412, 0.517)	0.034 (0.025, 0.046)	7.281 (6.321, 8.386)	-	13695.46
Negative binomial distribution + first-order	0.454 (0.390, 0.527)	0.036 (0.027, 0.049)	7.374 (6.256, 8.692)	-	13350.65
Negative binomial distribution + second-order	0.443 (0.384, 0.511)	0.025 (0.015, 0.041)	8.143 (6.723, 9.862)	-	13559.64
Negative binomial distribution + Power law	0.446 (0.382, 0.521)	0.178 (0.133, 0.237)	7.069 (5.999, 8.329)	-	13292.54
Negative binomial distribution + Power law + cov(PI)	0.446 (0.382, 0.520)	0.176 (0.131, 0.236)	11.540 (2.219, 59.983)	-0.075 (-0.327, 0.177)	13294.20
Negative binomial distribution + Power law + cov(MeanTemp)	0.446 (0.382, 0.521)	0.168 (0.122, 0.232)	17.680 (6.284, 49.762)	-0.543 (-1.151, 0.066)	13291.63
Negative binomial distribution + Power law + cov(MaxTemp)	0.447 (0.383, 0.522)	0.171 (0.125, 0.234)	15.830 (4.662, 53.740)	-0.438 (-1.099, 0.223)	13292.90
Negative binomial distribution + Power law + cov(MinTemp)	0.446 (0.382, 0.520)	0.173 (0.127, 0.235)	10.350 (4.790, 22.382)	-0.251 (-0.748, 0.247)	13293.60
Negative binomial distribution + Power law + cov(M_gs)	0.444 (0.380, 0.518)	0.180 (0.135, 0.240)	1.113 (0.548, 2.259)	1.072 (0.686, 1.458)	13263.96
Negative binomial distribution + Power law + cov(Max_gs)	0.441 (0.377, 0.515)	0.186 (0.140, 0.246)	0.552 (0.203, 1.503)	1.162 (0.721,1.602)	13266.44
Negative binomial distribution + Power law + cov(Desn)	0.446 (0.382, 0.520)	0.176 (0.131, 0.236)	11.540 (2.219, 59.983)	-0.075 (-0.327, 0.177)	13294.20
Negative binomial distribution + Power law + cov(GDP)	0.454 (0.389, 0.530)	0.173 (0.125, 0.239)	0.225 (0.108, 0.469)	0.823 (0.661, 0.984)	13185.85
Negative binomial distribution + Power law + cov(RH)	0.449 (0.384, 0.523)	0.176 (0.131,0.238)	0.954 (0.206, 4.418)	0.486 (0.118, 0.854)	13287.63
Negative binomial distribution + Power law + cov(SH)	0.446 (0.382, 0.521)	0.177 (0.133, 0.237)	7.343 (4.064, 13.267)	-0.027 (-0.425, 0.371)	13294.52
Negative binomial distribution + Power law + cov(WS)	0.447 (0.383, 0.521)	0.180 (0.135, 0.240)	5.050 (3.431, 7.433)	0.336 (-0.006, 0.677)	13290.77
Negative binomial distribution + Power law + cov(GDP) + cov(MeanTemp) + cov(Max_gs)	0.451 (0.386, 0.527)	0.187 (0.138, 0.252)	0.033 (0.005, 0.202)	0.804 (0.628, 0.981) 0.368 (-0.307, 1.043) 0.623 (0.189, 1.057)	13180.65

PI means proportion of primary industry; MeanTemp means weekly mean temperature; MaxTemp means weekly maximum temperature; MinTemp means the weekly minimum temperature; M_gs means mean weekly speed of gustiness; Max_gs means weekly maximum speed of gustiness; Desn means population density (/100,000); GDP means Real Gross Domestic Product (GDP) per capita; RH means weekly mean relative humidity; SH means mean weekly hours of sunshine; WS means mean weekly wind speed

*CI* Confidence interval, *AIC* Akaike information criterion

The total effect value of the autoregressive component was 0.451 (95% confidence interval (CI): 0.386,0.527), surpassing both the epidemic component (0.187, 95%CI: 0.138, 0.252) and endemic component (0.033, 95%CI: 0.005, 0.202) (Table 1), suggesting that the occurrence of HFRS in Shandong Province was primarily influenced by the autoregressive factor. The estimated effects of GDP per capita, weekly mean temperature and maximum speed of gustiness were 0.804 (95%CI: 0.628, 0.981), 0.368 (95%CI: -0.307, 1.043) and 0.623 (95%CI: 0.189, 1.057), respectively, demonstrating that higher GDP per capita, higher weekly mean temperature and higher maximum speed of gustiness prompted the transmission of HFRS. In all specifications, only GDP per capita and maximum speed of gustiness demonstrated statistical significance (Table 1).

**Fig. 5:**
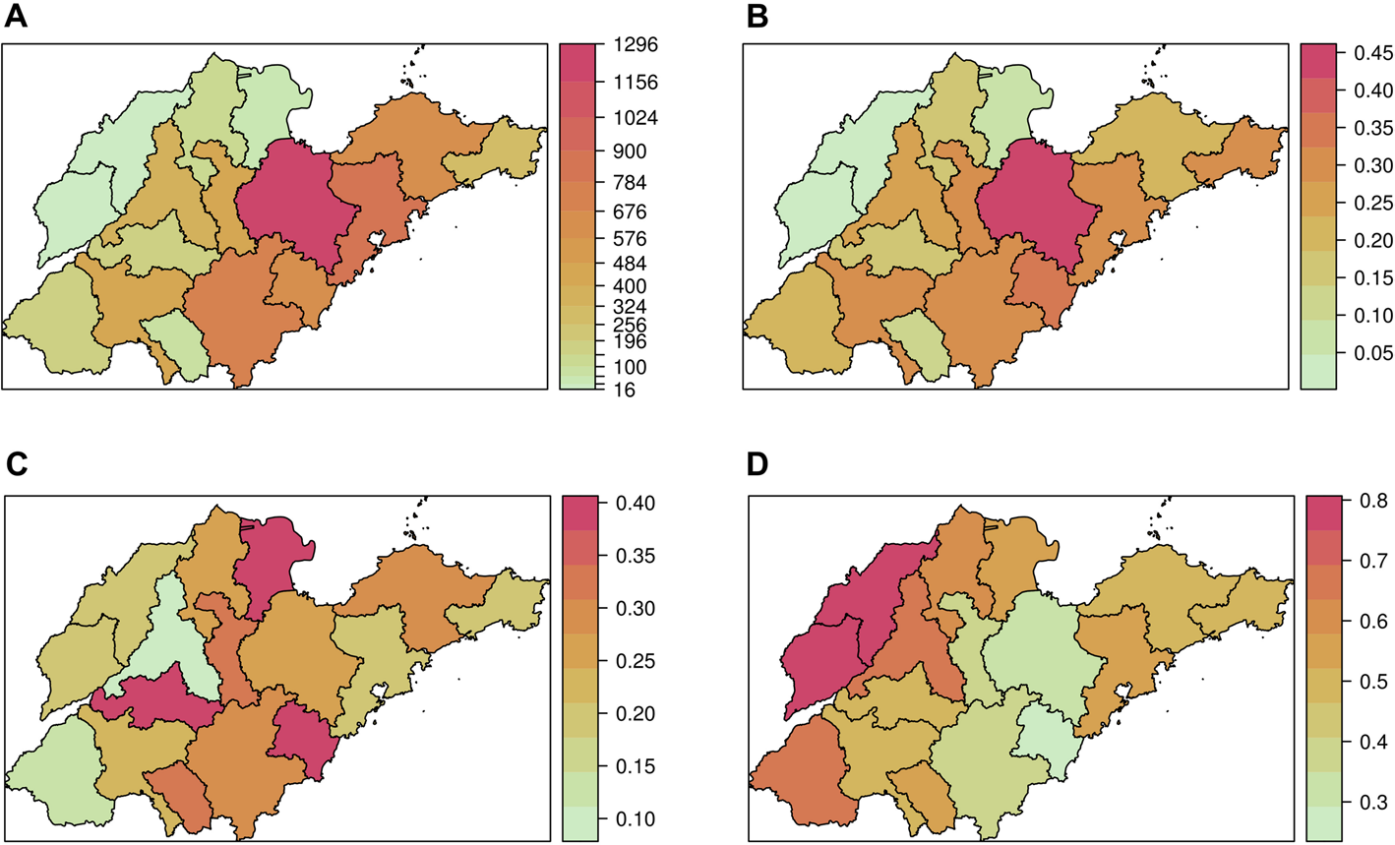
The district-specific total incidence and fitted component of HFRS in Shandong Province, China, 2016–2022. **A** The total case numbers of HFRS from 2016 to 2022. **B** The autoregressive component at the city level. **C** The epidemic component at the city level. **D **The endemic component at the city level

**Fig. 6:**
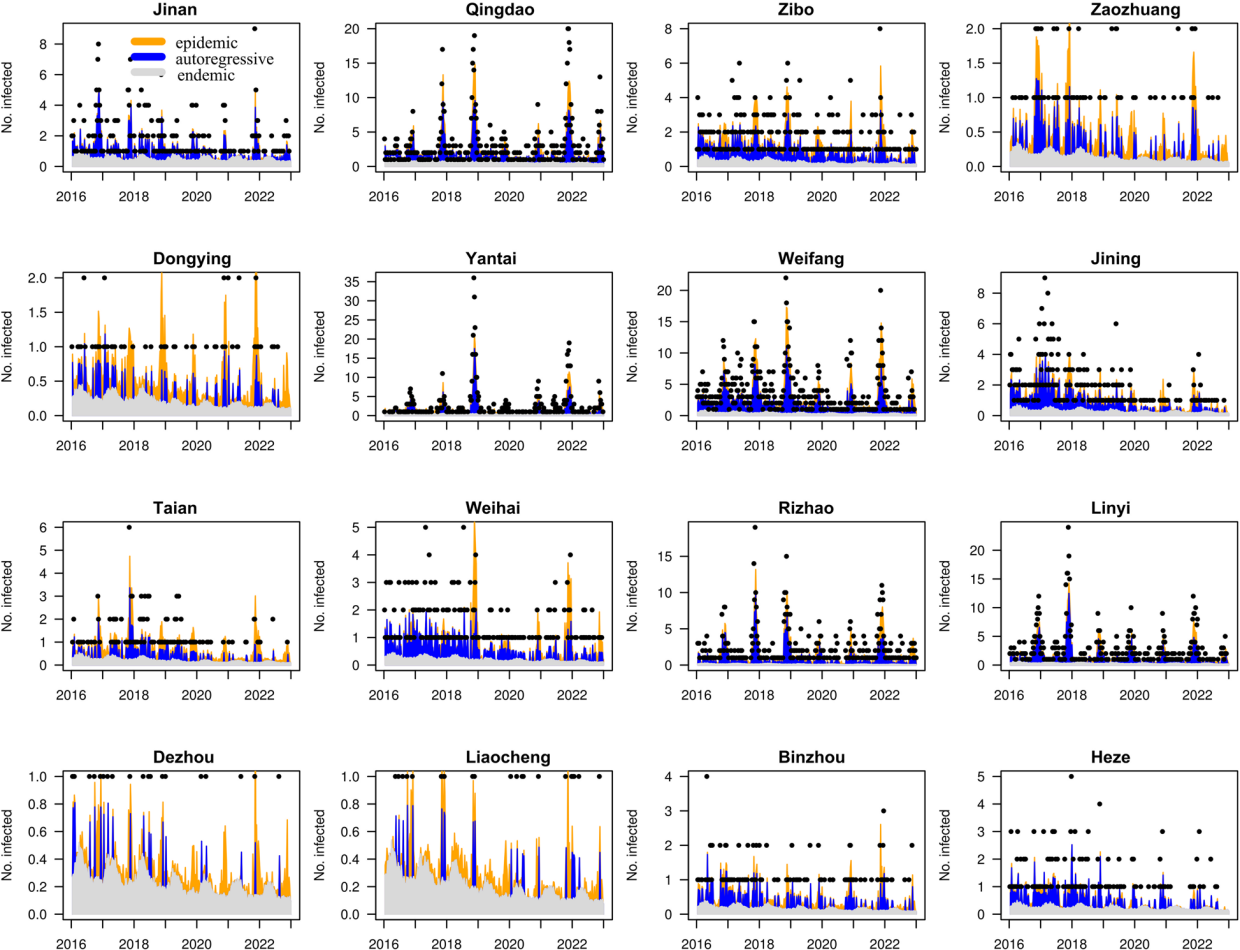
Fitted components in the spatial-temporal multicomponent model of sixteen cities in Shandong Province, China, 2016–2022

The mean values of the three components exhibited significant heterogeneity across cities (Fig. 5). In terms of the proportion of autoregressive component, the northern regions consistently demonstrated higher values than the southern regions, aligning with the incidence of HFRS. Notably, Weifang, a city with high incidence rate of HFRS, showed the highest proportion of autoregressive component compared to other cities (Fig. 5; Table 2). The distribution of the epidemic component appeared random, in contrast to the pattern of HFRS incidence (Figs. 2 and 5). Dongying, a city with a low incidence of HFRS, exhibited high proportion of epidemic and endemic components, indicating that these two components significantly drove the occurrence of HFRS in Dongying. The absolute values of the top epidemic components were lower than the values of the autoregressive component (Table 2). The proportions of the endemic component were similar to those of the epidemic component, characterized by higher values corresponding to lower HFRS incidence (Figs. 2 and 5). Notably, the Weihai epidemic in late 2018 was strongly influenced by the epidemic component, implying that cases in this area may have been imported from neighboring areas (Fig. 6). Another intriguing result was the negligible impact of the epidemic component at the beginning of 2020 (Fig. 6). In comparison to the earlier period, the epidemic component of Qingdao, Weifang, Weihai, and Jining sharply declined. Conversely, the endemic component prevailed in these cities.

**Table 2:** The mean values of the components of 16 cities in Shandong Province, 2016–2022

City	Autoregressive component	Epidemic component	Endemic component
Jinan^a^	0.389	0.100	0.100
Qingdao	0.912	0.524	0.524
Zibo	0.429	0.359	0.359
Zaozhuang	0.090	0.158	0.158
Dongying	0.061	0.221	0.221
Yantai	0.757	0.579	0.579
Weifang	1.314	0.773	0.773
Jining	0.428	0.263	0.263
Taian	0.164	0.265	0.265
Weihai	0.290	0.203	0.203
Rizhao	0.685	0.491	0.491
Linyi	0.817	0.479	0.479
Dezhou	0.022	0.081	0.081
Liaocheng	0.023	0.078	0.078
Biznhou	0.130	0.120	0.120
Heze	0.193	0.060	0.060

^a^including Laiwu city

The time-varying effects of the three components differed considerably in Shandong Province, with clear evidence of seasonality, particularly for the autoregressive and epidemic components. Throughout the study period, Qingdao, Yantai, Weifang, Rizhao, and Linyi were primarily influenced by the autoregressive component (Fig. 6). In Jinan and Heze, the incidences of HFRS were driven by both autoregressive and endemic components simultaneously. The endemic component emerged as the primary driving factor for Dezhou and Liaocheng. In Zibo, the epidemic component played a predominant role at the end of each year. The endemic component showed a declining trend in Dongying across all periods.

**Fig. 7:**
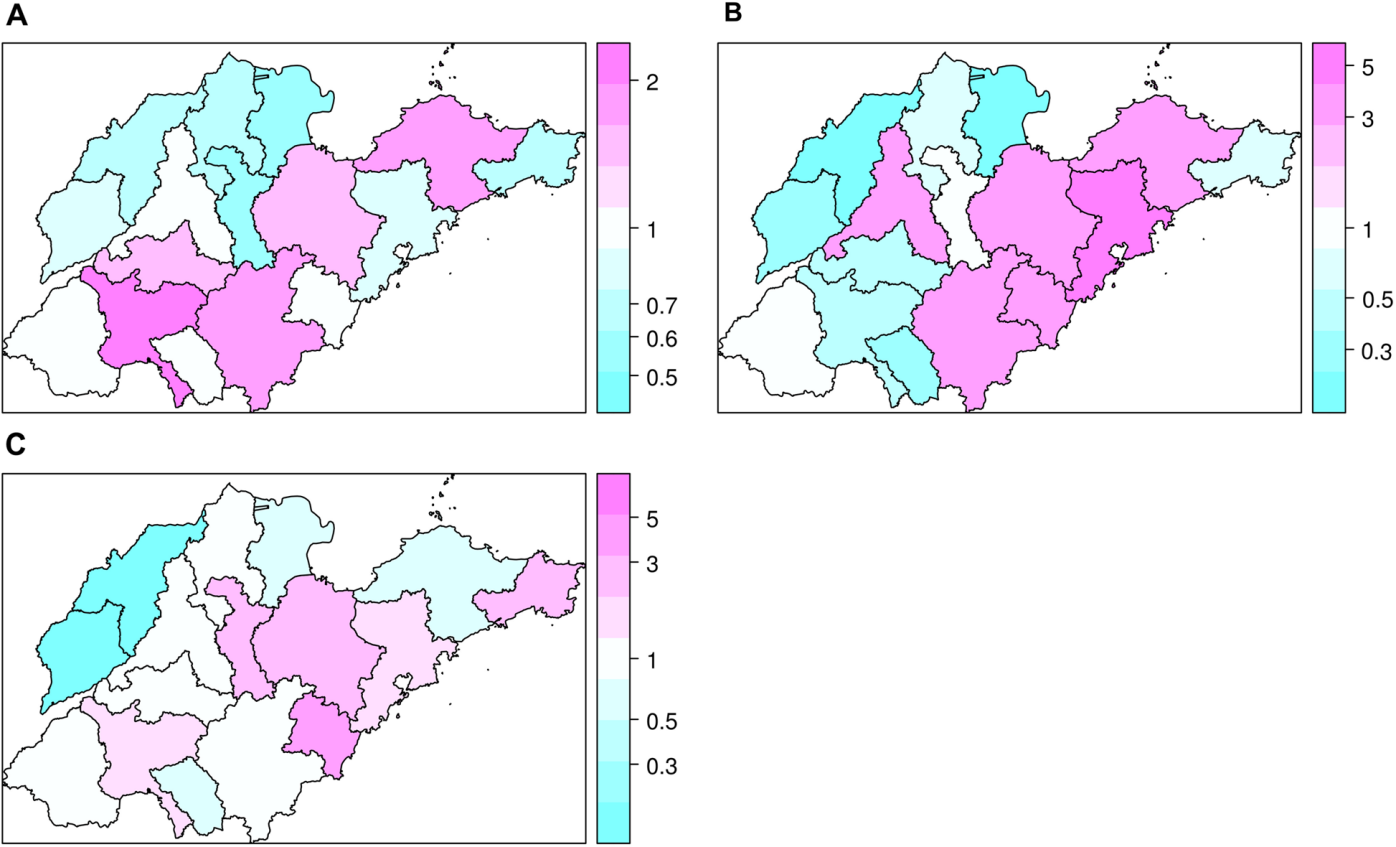
Maps of the estimated random intercepts. **A** Random effect for the autoregressive component. **B** Random effect for the epidemic component. **C** Random effect for the endemic component

Several cities exhibited disparate incidence levels that were not adequately explained by the observed covariates. For instance, Qingdao, characterized by high GDP per capita and a similar climatic environment to other HFRS epidemic areas (Yantai, Weihai, Weifang and Rizhao), displayed a low incidence rate of HFRS, whereas Weifang exhibited a high incidence rate. To account for this residual heterogeneity, we incorporated random effects into the spatial-temporal multicomponent model. As depicted in Fig. 7, Qingdao is represented in dark pink, signifying that it imports more cases from other regions than can be explained by the covariates (Fig. 7; Table 3). Weifang was influenced by local outbreaks and neighboring regions (Fig. 7; Table 3).

**Table 3:** The values of random intercept of components among cities in Shandong Province, 2016–2022

City	Autoregressive component	Epidemic component	Endemic component
Jinan^a^	0.921	3.194	0.876
Qingdao	0.754	4.732	1.316
Zibo	0.517	0.973	2.940
Zaozhuang	1.052	0.353	0.757
Dongying	0.523	0.233	0.699
Yantai	1.826	3.870	0.716
Weifang	1.550	3.207	2.822
Jining	2.382	0.539	1.462
Taian	1.446	0.452	0.895
Weihai	0.661	0.689	3.189
Rizhao	1.076	3.136	3.690
Linyi	1.941	3.272	1.043
Dezhou	0.640	0.160	0.166
Liaocheng	0.812	0.291	0.122
Biznhou	0.707	0.772	0.968
Heze	1.022	1.076	0.876

^a^including Laiwu city

## Discussion

As a prototypical natural focal disease, HFRS continues to pose a significant public health threat in China. Through the synthesis of surveillance, climatic and socioeconomic data spanning from 2016 to 2022 in Shandong Province, our study offered an update, novel, and in-depth analysis of the factors driving HFRS transmission. In areas with high incidence, the autoregressive component emerged as a key drive of HFRS transmission. Substantial heterogeneity was observed in the components across different cities. This study provided a distinctive perspective on the transmission pattern of HFRS, emphasizing the imperative for the government to develop targeted measures in response to the observed variations.

Via visual analysis, we observed a gradual increase in the HFRS epideic, reaching its peak in 2017 and subsequently decreasing from 2019, a trend parallel to the national epidemic situation [18]. This pattern can be attributed to several factors: 1) Increased attention to HFRS and a rise in vaccination coverage in recent years [19]; 2) Growing levels of urbanization and improved sanitary conditions, particularly in rural areas, reducing the suitable habitat for rodents. It is noteworthy that the early stages of urbanization have been linked to the emergence of HFRS [20]; 3) Strengthened infection prevention and control measures; 4) Robust intervention measures for COVID-19, such as travel restrictions, lockdowns, and the suspension of work and production, potentially curbing the spread of other infectious diseases [21]. Our findings indicated that central and eastern regions, encompassing the entire coastal cities of Shandong Province, were the most vulnerable. These areas, characterized by mountains, significant topographic fluctuations, and abundant precipitation, created favorable conditions for the survival of rodents [22]. Additionally, the high involvement of farmers in farming, breeding, and other agricultural activities in these regions elevates the risk of HFRS infection [23]. Intriguingly, a previous study highlighted “distance to coastline” as one of the crucial explanatory variables in the climate-HFRS association [24]. This underscores the importance of prioritizing the implementation of prevention and control measures in these regions. The seasonal profile of HFRS has been reported in different countries [25, 26]. We surmised that etiological factors contributed to this difference in the amplitude of peak activities. In China, HFRS cases are primarily associated with the causative pathogens HTNV and Seoul virus (SEOV). HTNV infections can occur throughout the year, with a majority reported in fall and winter, while SEOV is predominantly reported in the spring [22]. The carriers and transmitters of these viruses are commonly Apodemus agrarius, Mus musculus, and Rattus norvegicus [27]. Considering the living environment of rodents and the high-risk populations for HFRS infection [22, 23, 24, 25], we recommend the following prevention and control strategy in the endemic area: implementing measures for rodent prevention and extermination, monitoring rodent density and infection status, enhancing vaccination coverage, providing health education for individuals aged 60 and above, and intensifying environmental improvement activities.

Our study revealed a positive association between GDP per capita and the incidence of HFRS [28]. Economic growth reflects urbanization. Tian et al. observed a strong positive correlation between HFRS incidence and urbanization in the early stage of this process [20]. The primary factors contributing to this association include farmland conversion, deforestation, and other land use changes induced by urban expansion, leading to habitat loss and fragmentation that drive rodent movement and increase human exposure to rodents [20]. In China, Shandong Province is currently undergoing rapid urbanization characterized by mass rural-urban migration alongside swift urban expansion with underdeveloped infrastructure [29], contributing to a heightened risk of human infection. Unfortunately, urbanization rates were not included in our model due to lack of data. In short, developing regions should focus their attention on zoonotic diseases. Based on epidemiological evidence, areas with the highest risk of human infection are characterized by increased migration, crowded working and living conditions, especially in poor housing conditions, where close contact with the excreta of infected rodents is frequent. These areas require special attention. Additionally, vaccination programs need to take into account the particular challenge of human migration from rural to urban settings. The implementation of rigorous vaccination campaigns is essential for controlling HFRS. It is crucial to establish stringent standards and regulations to promote the clinical diagnosis and reporting of HFRS, as well as to build a well-trained healthcare workforce. HFRS is considered a climatic-sensitive infectious disease [30]. The impact of climatic drivers on HFRS incidence is complex and diverse, varying with region and magnitude [9, 10, 24, 27]. Temperature may influence HFRS dynamics through its impact on rodent reservoir population and pathogen survival in external environment, subsequently shaping patterns of human-animal contact [27, 31]. However, conflicting findings on the temperature effect make it challenging to draw definitive conclusions. In our study, no significant relationship between various temperatures and HFRS occurrence was observed. The inconsistent results may arise from variations in modeling methods, differences in regional climate, and geographical environments, especially environmental factors directly impacting the frequency of contacts between rodents and between rodents and humans, as well as the survival of the virus. More research is needed to investigate the mechanisms underlying these relationships.

HFRS transmission involves multiple routes, and its relationship with environmental, meteorological, or socioeconomic factors is intricate [5, 6, 9, 10, 20, 21, 23]. Combinations of these factors can lead to diverse patterns of HFRS transmission, causing variations in epidemic characteristics across cities and even provinces. Utilizing the spatial-temporal multicomponent model, we captured apparent spatial heterogeneity in the components driving HFRS transmission. The smaller value of the endemic component compared to the autoregressive and epidemic components suggests that factors such as climatological changes and genetic susceptibility may be similar among cities [16]. In this study, the autoregressive component played a key role in driving the transmission of HFRS, signifying that the epidemic effects from the previous season continuously contributed to the later peaks. Consequently, we recommend enhancing early-warning and emergency response to HFRS epidemics to reduce the risk of subsequent development, particularly in areas with high population density.

In comparison to other areas, Dezhou and Liaocheng, the cities minimally affected by HFRS, were predominantly by the endemic component, indicating that HFRS circulates within these two cities as an endemic disease. Therefore, targeted and comprehensive interventions, such as rodent control, health education, environmental management, improvement of living standards, and vaccination, may effectively reduce the number of HFRS cases. The transmission drivers of HFRS in Dongying and Zaozhuang were complex, reflecting the combined effects of the three components over the study period. This emphasized the importance of enhancing the surveillance capabilities for HFRS in these two cities, enabling the government to formulate preventive policies and practical solutions to address the evolving scenarios.

Infectious disease transmission is influenced by various factors, including gender, age, individual genetic variation, vaccination status, and the socioeconomic and meteorological factors mentioned above [32]. Undoubtedly, our model cannot include all significant factors due to limited knowledge and data availability, potentially resulting in heterogeneous incidence levels not explained by observed covariates. The epidemics of Qingdao and Weifang exhibited similar levels of heterogeneous. Paul and Held introduced random effects for the spatial-temporal multicomponent model, proving to be an effective approach to solve this problem [32]. Random effects accounted for the heterogeneous incidence levels in Qingdao and Weifang. After adjusting for socioeconomic and meteorological factors, Qingdao exhibited a relatively low endemic incidence, suggesting a potential under-reporting issue. For Weifang, the high number of cases may be explained by transmission from neighboring regions, subsequently establishing an endemic presence within the city.

Our study is subject to a few limitations. Firstly, all cases in our study were obtained from a passive surveillance, implying potential underreporting of HFRS cases. Secondly, due to insufficient data, crucial factors such as the density and virus-carrying rate of rodents were not included. Thirdly, our model did not consider the possibility that the initial detection of HFRS cases in some locations could be attributed to improved diagnosis rather than recent disease spread.

## Conclusions

In conclusion, our study presented the recent epidemiological characteristics and identified the main driving components of transmission, along with heterogeneous incidence levels among different cities in Shandong Province, China. We found that the autoregressive component played a pivotal role in driving HFRS transmission in Shandong Province. Significant heterogeneity in incidence levels and spatial-temporal components was observed across all cities, providing specific insights for the development of targeted interventions. Sequels to this study must endeavour to improve spatial resolution and focus on a better understanding of the reasons behind spatial-temporal incidence heterogeneity of HFRS.

### Supplementary Information

**Supplementary Material 1.**

## Data Availability

The weekly reported cases used for analysis is available to the public via supplementary Table S1. The daily meteorological data for cities were sourced from the China Meteorological Data Sharing Service System (http://data.cma.cn/). The city-level socioeconomic data were collected from the Shandong Statistical Yearbook (http://tjj.shandong.gov.cn/col/col6279/index.html).
